# Publisher Correction: Multimodal cell maps as a foundation for structural and functional genomics

**DOI:** 10.1038/s41586-025-09648-x

**Published:** 2025-10-02

**Authors:** Leah V. Schaffer, Mengzhou Hu, Gege Qian, Kyung-Mee Moon, Abantika Pal, Neelesh Soni, Andrew P. Latham, Laura Pontano Vaites, Dorothy Tsai, Nicole M. Mattson, Katherine Licon, Robin Bachelder, Anthony Cesnik, Ishan Gaur, Trang Le, William Leineweber, Aji Palar, Ernst Pulido, Yue Qin, Xiaoyu Zhao, Christopher Churas, Joanna Lenkiewicz, Jing Chen, Keiichiro Ono, Dexter Pratt, Peter Zage, Ignacia Echeverria, Andrej Sali, J. Wade Harper, Steven P. Gygi, Leonard J. Foster, Edward L. Huttlin, Emma Lundberg, Trey Ideker

**Affiliations:** 1 Department of Medicine, University of California San Diego, La Jolla, CA USA; 2 Bioinformatics and Systems Biology Program, University of California San Diego, La Jolla, CA USA; 3 Department of Biochemistry & Molecular Biology, Michael Smith Laboratories, University of British Columbia, Vancouver, British Columbia Canada; 4 Department of Bioengineering and Therapeutic Sciences, University of California San Francisco, San Francisco, CA USA; 5 Department of Cell Biology, Harvard Medical School, Boston, MA USA; 6 Department of Bioengineering, Stanford University, Palo Alto, CA USA; 7 Broad Institute of MIT and Harvard, Boston, MA USA; 8 Department of Pediatrics, Division of Hematology-Oncology, University of California San Diego, La Jolla, CA USA; 9 Department of Cellular and Molecular Pharmacology, University of California San Francisco, San Francisco, CA USA; 10 Quantitative Biosciences Institute, University of California San Francisco, San Francisco, CA USA; 11 Department of Pharmaceutical Chemistry, University of California San Francisco, San Francisco, CA USA; 12 Department of Pathology, Stanford University, Palo Alto, CA USA; 13 Science for Life Laboratory, School of Engineering Sciences in Chemistry, Biotechnology and Health, KTH Royal Institute of Technology, Stockholm, Sweden; 14 Chan Zuckerberg Biohub, San Francisco, CA USA; 15 Department of Computer Science and Engineering, University of California San Diego, La Jolla, CA USA; 16 Department of Bioengineering, University of California San Diego, La Jolla, CA USA

**Keywords:** Data integration, Machine learning, Network topology, Proteome informatics

Correction to: *Nature* 10.1038/s41586-025-08878-3 Published online 9 April 2025

In the version of the article initially published, the fourth equation in the “Loss functions” section of the Methods was a duplicate of the fifth equation. In the fourth equation, “*T*_*y*_” and both instances of “*S*_*y*_” have been corrected to “*T*_*x*_” and “*S*_*x*_” so that the equation now reads:

$${T}_{x}=\frac{1}{m}\sum _{i\varepsilon N}\sum _{j\varepsilon N\,,j\ne i}\sum _{k\varepsilon N\,,k\ne i\,,j}{S}_{x}(i\,,j)(1\,-\,{S}_{x}(i,k))\times {\rm{\text{max}}}(D({{\bf{z}}}_{i},{{\bf{z}}}_{j})-D({{\bf{z}}}_{i},{{\bf{z}}}_{k})+\varepsilon ,0)$$

in the HTML and PDF versions of the article.

