# Supplementary material for: Multimodal cell maps as a foundation for structural and functional genomics
Source: Nature. 2025 Apr 9;642(8066):222–31. doi: 10.1038/s41586-025-08878-3 (PMC12137143; doi:10.1038/s41586-025-08878-3)
Supplement: Supplementary file 1 [file 41586_2025_8878_MOESM2_ESM.pdf]

nature portfolio

Corresponding author(s): Trey Ideker, Emma Lundberg, Edward L. Huttlin

Last updated by author(s): January 31, 2025

Reporting Summary

Nature Portfolio wishes to improve the reproducibility of the work that we publish. This form provides structure for consistency and transparency in reporting. For further information on Nature Portfolio policies, see our Editorial Policies and the Editorial Policy Checklist.

Statistics

For all statistical analyses, confirm that the following items are present in the figure legend, table legend, main text, or Methods section.

n/a	Confirmed	
☐	☒	The exact sample size (*n*) for each experimental group/condition, given as a discrete number and unit of measurement
☐	☒	A statement on whether measurements were taken from distinct samples or whether the same sample was measured repeatedly
☐	☒	The statistical test(s) used AND whether they are one- or two-sided<br>*Only common tests should be described solely by name; describe more complex techniques in the Methods section.*
☒	☐	A description of all covariates tested
☐	☒	A description of any assumptions or corrections, such as tests of normality and adjustment for multiple comparisons
☐	☒	A full description of the statistical parameters including central tendency (e.g. means) or other basic estimates (e.g. regression coefficient) AND variation (e.g. standard deviation) or associated estimates of uncertainty (e.g. confidence intervals)
☐	☒	For null hypothesis testing, the test statistic (e.g. *F*, *t*, *r*) with confidence intervals, effect sizes, degrees of freedom and *P* value noted<br>*Give P values as exact values whenever suitable.*
☒	☐	For Bayesian analysis, information on the choice of priors and Markov chain Monte Carlo settings
☐	☒	For hierarchical and complex designs, identification of the appropriate level for tests and full reporting of outcomes
☐	☒	Estimates of effect sizes (e.g. Cohen's *d*, Pearson's *r*), indicating how they were calculated

Our web collection on statistics for biologists contains articles on many of the points above.

Software and code

Policy information about availability of computer code

Data collection

Immunofluorescence confocal images were collected using 63x oil immersion with Numerical Aperture 1.4. AP-MS data were acquired on first-generation Q-Exactive mass spectrometers (Thermo Fisher Scientific) equipped with Famos autosamplers (LC Packings) and Accela600 liquid chromatography (LC) pumps (Thermo Fisher Scientific). SEC-MS data was acquired on a TimsTOF Pro2 (Bruker Daltonics) with CaptiveSpray source coupled to nanoElute UHPLC (Bruker Daltonics).

Data analysis

All data analyses have been described in detail in the relevant Methods section with links to publicly available GitHub repositories. The cell map construction pipeline is available at https://github.com/idekerlab/cellmaps_pipeline. The required Python packages, along with specific versions, were documented in GitHub.

The following versions were used to construct the cell map:
Python== 3.8.16
cellmaps_imagedownloader==0.1.0a7
cellmaps_ppidownloader==0.1.0a6
cellmaps_image_embedding==0.1.0a7
cellmaps_ppi_embedding==0.2.0a6
cellmaps_coembedding==0.1.0a6
cellmaps_generate_hierarchy==0.1.0a13
cellmaps_hierarchyeval==0.1.0a5

The following versions were used to analyze the cell map (Use Cases):
Python==3.7.9

scipy==1.7.3

The following versions were used for the Random Forest analysis:
Python==3.9.18
scikit-learn==1.3.0

Other softwares used in this study are documented in the Methods section and listed below:
CompPASS https://github.com/dnusinow/cRomppass
CompPASS-Plus https://github.com/HMSBioPlex/ CompPASS-Plus-CLI
Cytoscape v3.10.1
DIA-NN 1.8.1.0 https://github.com/vdemichev/DiaNN
HiSig https://github.com/fanzheng10/HiSig
IUPred3.0 https://iupred3.elte.hu/download_new
PrinCE https://github.com/fosterlab/PrInCE
localcolabfold https://github.com/YoshitakaMo/localcolabfold
Integrative Modeling Platform (IMP) package v2.18 https://integrativemodeling.org
MutSigCV v1.4 (available in https://github.com/fanzheng10/HiSig/)

For manuscripts utilizing custom algorithms or software that are central to the research but not yet described in published literature, software must be made available to editors and reviewers. We strongly encourage code deposition in a community repository (e.g. GitHub). See the Nature Portfolio guidelines for submitting code & software for further information.

Data

Policy information about availability of data

All manuscripts must include a data availability statement. This statement should provide the following information, where applicable:

- Accession codes, unique identifiers, or web links for publicly available datasets
- A description of any restrictions on data availability
- For clinical datasets or third party data, please ensure that the statement adheres to our policy

The Multiscale Integrated Cell web portal (musicmaps.ai/u2os-cellmap) provides links to all major data and derived resources associated with this study, including AP-MS protein interactions, protein immunofluorescence images, size-exclusion chromatography data, and the online interactive U2OS cell map. The U2OS cell map is available on ndexbio.org with the uuid f693137a-d2d7-11ef-8e41-005056ae3c32. Protein assemblies in the cell map are also available at the European Bioinformatics Institute (EBI) Protein Complex Portal (https://www.ebi.ac.uk/complexportal) with the query CLO:0009454. The AP-MS protein interactions are available on ndexbio.org with the uuid 95bc75d5-d1d1-11ee-8a40-005056ae23aa. In addition to its release here, the U2OS protein interaction network will be included as part of the upcoming BioPlex v4.0 database release (Huttlin et al. in preparation). AP-MS raw mass spectrometry files are available on MassIVE with the identifier MSV000097168. The entire image dataset is included in the Human Protein Atlas v23 release. SEC-MS raw mass spectrometry files and search results are available via Proteome Xchange with the identifier PXD052362. All structural models are available in the ModelArchive Database (modelarchive.org) with the identifiers ma-idk-u2osmap and ma-m5og4. Other public databases and resources used in this study include the Gene Ontology (June 2023 release, https://geneontology.org), CORUM (version 4.1 release, https://mips.helmholtz-muenchen.de/corum/), UniProt Homo sapiens proteome (accessed June 2 and September 11, 2023, https://uniprot.org), STRING interactome (v12, NDEx uuid 0b04e9eb-8e60-11ee-8a13-005056ae23aa), OpenCell interactions (https://opencell.czbiohub.org/download), CD-CODE condensate database (accessed May 31, 2023, https://cd-code.org), FuzDrop (Dataset S7 in Hardenberg et al.), Protein Condensate Atlas (Supplementary Dataset 8 in Saar et al.) K562 day-8 perturb-seq dataset (gwps.wi.mit.edu), HEK-293 BioPlex v3.0 (NDEx uuid 6b995fc9-2379-11ea-bb65-0ac135e8bacf), pediatric cancer mutation data (https://www.cbioportal.org/study/summary?id=pediatric_dkfz_2017), and transposon-based mutagenesis screens from the Candidate Cancer Gene Database (http://ccgd-starrlab.oit.umn.edu/index.html, downloaded March 26, 2024).

Research involving human participants, their data, or biological material

Policy information about studies with human participants or human data. See also policy information about sex, gender (identity/presentation), and sexual orientation and race, ethnicity and racism.

Reporting on sex and gender	Not applicable
Reporting on race, ethnicity, or other socially relevant groupings	Not applicable
Population characteristics	Not applicable
Recruitment	Not applicable
Ethics oversight	Not applicable

Note that full information on the approval of the study protocol must also be provided in the manuscript.

Field-specific reporting

Please select the one below that is the best fit for your research. If you are not sure, read the appropriate sections before making your selection.

☒ Life sciences ☐ Behavioural & social sciences ☐ Ecological, evolutionary & environmental sciences

For a reference copy of the document with all sections, see nature.com/documents/nr-reporting-summary-flat.pdf

Life sciences study design

All studies must disclose on these points even when the disclosure is negative.

Sample size	The number of proteins analyzed in this study (n=5147) was determined based on amount of matched data available when overlapping immunofluorescence image and AP-MS interactions in the U-2 OS cell line. For follow-up experiments in this study, no statistical methods were used to pre-determine sample sizes, which were chosen to reliably observe experimental phenotypes.
Data exclusions	No data were excluded from analyses
Replication	All the data collected in this study consisted of technical or biological replicates. The number of replicates, as well as the type of replicates (i.e. technical or biological), are labeled in the relevant figures or method sections.
Randomization	AP-MS baits were arrayed on 96-well plates in random order, and plates were run in random order during LC-MS analysis. For other experiments in this study, randomization was used whenever possible to determine experimental order.
Blinding	All IF, AP-MS, and SEC-MS data were generated and processed with investigators blinded to the hypothesis.<br>For DPP9 RT-qPCR measurement (Extended Data Fig. 6c), blinding was not applied during analysis, which we followed established procedure from previous studies.

Reporting for specific materials, systems and methods

We require information from authors about some types of materials, experimental systems and methods used in many studies. Here, indicate whether each material, system or method listed is relevant to your study. If you are not sure if a list item applies to your research, read the appropriate section before selecting a response.

Materials & experimental systems

n/a	Involved in the study
☐	☒ Antibodies
☐	☒ Eukaryotic cell lines
☒	☐ Palaeontology and archaeology
☒	☐ Animals and other organisms
☒	☐ Clinical data
☒	☐ Dual use research of concern
☒	☐ Plants

Methods

n/a	Involved in the study
☒	☐ ChIP-seq
☒	☐ Flow cytometry
☒	☐ MRI-based neuroimaging

Antibodies

Antibodies used	The antibodies used in this study are listed as below. The dilutions used for each are specified in the "Matched protein immunofluorescence (IF) imaging data" section in the methods.<br>- Anti-tubulin Abcam, ab7291, RRID:AB_2241126<br>-Chicken anti-calreticulin, Abcam, ab14234, RRID:AB_2228460<br>- Rabbit polyclonal HPA antibodies, generated within the Human Atlas Project. The list of HPA antibody IDs used in this study are found at http://musicmaps.ai/u2os-cellmap.<br>-goat anti-rabbit Alexa488 A11034 , RRID:AB_2576217, RRID:AB_2535845, ThermoFisher, polyclonal<br>-goat anti-mouse Alexa555 A21424,RRID:AB_2535845, ThermoFisher, polyclonal<br>-goat anti-chicken Alexa647 A-21449, RRID:AB_2535866, ThermoFisher, polyclonal<br>-goat anti-rat Alexa647 A21247, ThermoFisher RRID:AB_1056356, polyclonal
Validation	All HPA antibodies were validated as described at https://www.proteinatlas.org/about/antibody+validation. The HPA antibodies are quality controlled for sensitivity and lack of cross-reactivity to other proteins using western blot and protein arrays. Antibodies that pass initial quality assessment are labeled as 'approved'. Antibodies that yield a staining pattern supported by independent data in UniProt are labeled as 'supported'. For 'enhanced' antibody validation, we use the strategies outlined by the International Working Group for Antibody Validation (IWGAV), including genetic validation, recombinant expression validation, independent antibody validation targeting a different epitope, and capture validation by mass spectrometry.

Eukaryotic cell lines

Policy information about cell lines and Sex and Gender in Research

Cell line source(s)	U-2 OS cells were obtained from the American Type Culture Collection (ATCC). HEK-293 data was published previously (Huttlin et al. Cell 2021)
Authentication	The U-2 OS cells used for IF stainings were authenticated according to the manufacturer ATCC using morphology, karyotyping and PCR based approaches to confirm the identity and to exclude intra and interspecies contaminations. These include an assay to detect species specific variants of the cytochrome C oxidase I gene (COI analysis) to rule out interspecies contamination and short tandem repeat (STR) profiling to distinguish between individual human cell lines and rule out intraspecies contamination. These cells were also used or the SEC-MS experiments and DPP9 experiments. The U-2 OS cells used for AP-MS were purchased directly from ATCC and no further authentication was performed.
Mycoplasma contamination	All cells used in this study were tested negative for mycoplasma contamination.
Commonly misidentified lines (See ICLAC register)	No commonly misidentified cell lines were used in this study.

Plants

Seed stocks	No seed stocks were used in this study.
Novel plant genotypes	No novel plant genotypes were produced in this study.
Authentication	Not applicable
